# Health status, care needs, and assessment for beneficiaries with or without dementia in a public long-term care insurance pilot in Guangzhou, China

**DOI:** 10.1186/s12913-020-05965-1

**Published:** 2020-12-07

**Authors:** Jialan Wu, Siman Chen, Huangliang Wen, Yayan Yi, Xiaoyan Liao

**Affiliations:** 1 Department of Nursing, Zengcheng Branch, Nanfang Hospital, Southern Medical University, No. 28 Innovation Avenue, Zengcheng, Guangzhou, 511300 China; 2 School of Nursing, Southern Medical University, No. 1838 Guangzhou Avenue North, Guangzhou, 510515 China

**Keywords:** Barthel index, Dementia, Eligibility, Long-term care insurance, Rasch analysis

## Abstract

**Background:**

Chinese government launched a pilot study on public long-term care insurance (LTCI) recently. Guangzhou is one of the fifteen pilot cities, officially started providing LTCI in August 2017. An in-depth analysis of experimental data from the pilot city may provide suggestions for developing a fair and effective LTCI system. This study aimed to evaluate the LTCI pilot by exploring the characteristics and care needs of claimants, and performance of the assessment tool.

**Methods:**

A retrospective cross-sectional study in which claims data between July 2018 and March 2019 in the Guangzhou pilot was analyzed. LTCI claimants during the study period were included. The care needs were determined based on claimants’ physical function assessed by the Barthel Index and their medical conditions. Rasch analysis was used to explore the performance of the Barthel Index.

**Results:**

Among 4810 claimants included, 4582 (95.3%) obtained LTCI benefits. Of these beneficiaries, 4357 (95.1%) were ≧ 60 years old, and 791 (17.3%) had dementia. Among 228 (4.7%) unsuccessful claimants, 22 (0.5%) had dementia. The prevalence of stroke was high in beneficiaries with (38.1%) or without dementia (56.6%), as well as in unsuccessful claimants with (40.9%) or without dementia (52.4%). Beneficiaries without dementia needed more support for basic activities of daily living and nursing care than those with dementia, while beneficiaries with dementia were more likely to be institutionalized. Five (22.7%) unsuccessful claimants with dementia and 48 (23.3%) unsuccessful claimants without dementia were disabled in at least two basic self-care activities. Regarding Barthel Index, Rasch analysis showed threshold disordering in “mobility” and “climbing stairs”, and the narrow interval was observed between all the adjacent categories of the ten items (< 1.4 logits).

**Conclusions:**

Stroke and dementia were two common reasons for needing long-term care in LTCI claimants. The Barthel Index is not suitable for assessing and dividing LTCI claimants, because of inappropriate items and narrow category responses. A comprehensive assessment and grading system is required, together with needs-led care services. The eligibility should be expanded gradually based on balance finance solutions.

**Supplementary Information:**

The online version contains supplementary material available at 10.1186/s12913-020-05965-1.

## Background

In China, it is estimated that approximately 167 million people were over 65 years old by the end of 2018, or 11.9% of the total population [1]. The growing demand for long-term care has become a huge challenge for the Chinese government. Aiming to establish a fair and effective national policy framework for public long-term care insurance by 2020, the government launched a pilot study on LTCI in 15 cities in June 2016 [2]. The National Healthcare Security Administration states that these pilots covered 88.54 million people by the end of June 2019, with 426,000 people receiving benefits [3]. An in-depth analysis of experimental data from pilot cities may help to understand the challenges faced by the pilot and provide suggestions for developing a more comprehensive LTCI system.

Guangzhou is one of the fifteen pilot cities located in south China. It officially started providing LTCI in August 2017 [4]. Guangzhou city had about 9,276,914 residents at the end of 2018, with 18.3% over 60 years old [5]. Of 1,692,692 residents of Guangzhou aged over 60 at the end of 2018, [5] 840,900 (49.7%) were insured [6]. Like other LTCI pilots in China, the Guangzhou pilot is financed by the Urban Employee Basic Medical Insurance scheme to reduce implementation difficulties and financial pressure [2]. Thus, the insured population comprises employees covered by the Urban Employee Basic Medical Insurance scheme in the pilot phase. According to the National Healthcare Security Administration, coverage of the LTCI will gradually expand to urban and rural residents covered by social medical insurance, and eventually achieve full coverage [3].

Guangzhou is one of three pilot cities expanding LTCI coverage to people with dementia [2]. However, these patients have to fulfill additional requirements to be eligible for LTCI benefits, including moderate to severe physical dependence and diagnosis of moderate or severe dementia. The restricted eligibility excludes people with mild dementia, or people with moderate-to-severe dementia but mild physical dysfunction. Our previous study found that 33.8% of people with moderate to severe dementia were not eligible for the benefits because of their high functional ability (Barthel Index > 60) [7]; Dementia affects 5–8% of those aged 65 and more, [8] and is one of the main reasons for requiring long-term care [9]. Alongside the rapid aging of the Chinese population, the population with dementia is projected to increase from 9.5 million in 2015 to 16 million by 2030 [8]. It is reasonable enough to ask whether the pilot has developed an appropriate way of allocating limited resources to particular vulnerable or at-risk populations, such as people with dementia.

Ten of the fifteen LTCI pilot cities use the Barthel Index to measure functional dependence [2]. While our previous study found that the Barthel Index is not suitable for assessing physical dependence in people with dementia based on Rasch analysis [10]. Policymakers should review the current assessment tool to determine whether it is an appropriate tool to assess and classify LTCI claimants. Rasch analysis, a probabilistic mathematical modeling technique based on item response theory, was developed to judge both the quality of the instrument and the latent trait (such as physical function) that an individual could possess. The advantage of Rasch analysis is that it can provide more interpretable information of the instrument in evaluating targeting populations.

Two years after the implementation of the pilot, it is helpful to consider whether the pilot has any flaws or loopholes that must be addressed. This study aimed to evaluate the LTCI pilot by exploring the traits of beneficiaries and unsuccessful claimants, their needs for long-term care services, and performance of the assessment tool, based on analysis of claims data from the Guangzhou pilot, and therefore provides suggestions for improving the LTCI system.

## Methods

### Data source

This retrospective study used claims data between July 2018 and March 2019 in the Guangzhou pilot, and assessment data required to determine eligibility for LTCI benefits. There were four main types of data: demographic information, physical function, medical conditions, and final decision about insurance benefits. All data were anonymized, with unique numbers assigned to each applicant. Before the researchers access to the dataset, personal information (such as name, address, post code, medical record number, medical insurance number, and social insurance number) of the applicants has been removed, together with any other information that could identify the applicants. The file contained the anonymized data has been kept in a locked place. ID has been used for data analysis. The Ethics Committee of Nanfang Hospital (NFEC-2020-187) approved this study and waived informed consent.

### Participants

Participants were extracted by panel sampling technique. All LTCI applicants in the Guangzhou pilot between July 2018 and March 2019 were included as the study population. The exclusion criteria were: (1) duplicated ID; (2) incomplete medical information; (3) beneficiaries with dementia lacking medical diagnosis of dementia.

### LTCI eligibility

When insured individuals request long-term care services, they are assessed for physical function, long-term care needs, health status, and/or diagnosis of dementia. This is reviewed by a committee of professional experts invited by the Guangzhou Healthcare Security Administration. A final decision is made within a week. Applicants who fail to pass the first assessment can be re-evaluated 15 days later. Claimants are re-evaluated every 12 months to confirm eligibility.

There are several eligibility criteria for the LTCI benefits of the pilot. First, insured people should have been functionally dependent for at least six months because of old age, chronic disease, or disability, but have a stable medical status. Second, functional disability was assessed using the Barthel Index. People without dementia should score 40 points or less. People with dementia should score 60 points or less and be diagnosed with moderate or severe dementia by a psychiatrist or neurological physician who works in an authorized hospital.

One or more forms of nursing treatment (such as stoma care, oxygen therapy, pressure sore care, dressing change, and catheterization care) are provided, based on the medical conditions of the claimants, including (1) long-term use of tracheal cannula, gastric tube, biliary tract drainage tubes, ostomy tube, urinary tube, deep vein tube, or other catheterization that needs regular treatment; (2) paralysis caused by disease or trauma, with a score on the Lovett scale for grading muscle strength of ≤3 for at least one lower limb, or moderate to severe dyskinesia (non-limb paralysis); or (3) vegetative state or cachexia, because they are in the end-stage of a chronic disease (e.g., malignant tumor) that requires long-term care [4].

### LTCI benefits

In the Guangzhou pilot, two types of in-kind services, institutional care and home care, are provided for beneficiaries of any age. Beneficiaries can choose the type of service they wish to receive, but there is no provision for a cash allowance. The co-payments are 25% for institutional care and 10% for home care. The upper limit on service expenses is CNY 120 per day for institutional care and CNY 115 per day for home care. Home care mainly focuses on basic activities of daily living (BADL) support and nursing care closely related to BADL (see Additional file 1). Nursing care expenses should not exceed 1000 CNY per month.

### Statistical analysis

Continuous data are shown as mean ± standard deviation, and categorical data as frequency and percentage. The Mann–Whitney U test was used for comparison of categorical data between groups and nonparametric data, using SPSS 20.0 (IBM SPSS Statistics, IBM Corporation, Chicago, IL). Bonferroni correction was used for multiple comparisons. Unsuccessful claimants without dementia were excluded from multiple comparisons, because of the small sample size. Rasch analysis based on the partial credit model was used to evaluate claimants’ traits and performance of the Barthel Index using WINSTEPS® 4.0 (SWREG Inc., USA).

To ensure that the Barthel Index data fit the Rasch model, we first assessed unidimensionality, global fit, and local fit of the data, and then mainly examined: (1) person separation (< 2, equal to person reliability < 0.8, implying narrow ability range of the samples); (2) targeting of the Barthel Index to claimants (mean person location should approximate to zero for a well-targeted tool); (3) threshold ordering of the categories and interval between the categories. A narrow distance between two adjacent categories (< 1.4 logits) indicated that the categories could be collapsed. These methods have been previously described [11]. Because large sample sizes (> 1000) might be considered to inflate the chi-square value and increase the likelihood of identifying misfit, [12] we conducted a sensitivity analyses by randomly selecting beneficiaries with (*n* = 300) or without dementia (*n* = 300), and unsuccessful claimants (*n* = 228) from the total sample, to validate results from the total sample. We also used the Wright person–item map to demonstrated distributions of item difficulties and claimants’ abilities.

The Markov model suggests that severe dependency refers to a state in which a person is dependent in at least one of six basic self-care activities (bathing, dressing, toilet use, transfer, continence and feeding) and needs help one or several times per day [13]. Therefore, we counted the number of disabilities in the six basic self-care activities for the participants according to the Markov model.

## Results

### Health status of the claimants

**Fig. 1:**
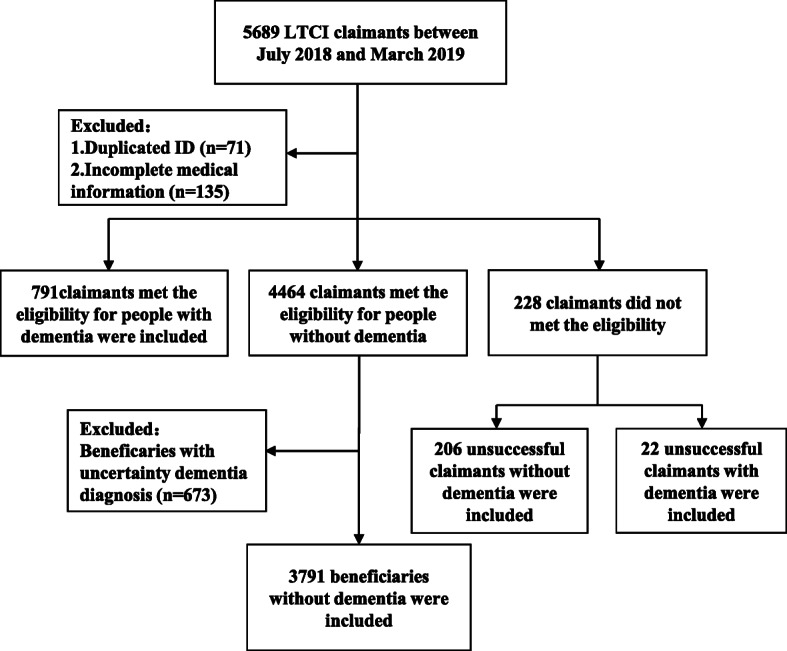
The participants inclusion flow chart

A total of 5689 LTCI claimants during the study period were included. Of these, 879 (15.4%) also met the exclusion criterion, leaving 4810 (84.6%) claimants eligible for inclusion. Figure 1 shows the participants inclusion flow chart. Table 1 shows **t**he demographic and clinical characteristics of the claimants included. Among 4810 claimants included, 4582 (95.3%) obtained benefits of LTCI. Of the 4582 beneficiaries, 225 (4.9%) were under 60 years old, 1170 (25.5%) were 60–80, 3187 (69.6%) were over 80, and 791 (17.3%) had dementia. Among 228 (4.7%) unsuccessful claimants, 22 (0.5%) had dementia. Beneficiaries with dementia were older than those without dementia. Of beneficiaries with dementia, 525 (66.4%) scored less than 40 for the Barthel Index. The unsuccessful claimants without dementia had better physical function (assessed by the Barthel Index) and muscle strength (assessed by the Lovett scale) than beneficiaries with or without dementia (Table 1).

**Table 1:** Demographic and clinical characteristics of the LTCI claimants (*n* = 4810)

Characteristics	Beneficiaries		Unsuccessful claimants	
	without dementia (***n*** = 3791)	with dementia (***n*** = 791)	without dementia (***n*** = 206)	with dementia (***n*** = 22)
Age (year), Mean (SD)	81.1 (11.8)	83.8 (8.4) ^a^	82.1 (10.8)	84.1 (8.0)
Female, n (%)	2452 (64.7)	544 (68.8)	124 (60.2)	16 (72.7)
BI score, Mean (SD)	18.2 (14.1)	30.4 (20.2) ^a^	62.1 (12.3) ^a,b^	76.1 (9.4)
LSGMS Lower ≤3, n (%)	3482 (91.8)	428 (54.1) ^a^	53 (25.7) ^a,b^	3 (13.6)
LSGMS Upper ≤3, n (%)	2836 (74.8)	324 (41.0) ^a^	32 (15.5) ^a,b^	2 (9.1)
**Care utilization, n (%)**				
Nursing home	2070 (54.6)	499 (63.1) ^a^	133 (64.6) ^a^	14 (63.6)
Home care	1721 (45.4)	292 (36.9)	73 (35.4)	8 (36.4)
**Chronic disease, n (%)**				
Hypertension	2460 (64.9)	430 (54.4) ^a^	144 (69.9) ^b^	16 (72.7)
Cerebrovascular disease	2195 (57.9)	311 (39.3) ^a^	123 (59.7) ^b^	12 (54.5)
Stroke	2147 (56.6)	301 (38.1) ^a^	108 (52.4) ^b^	9 (40.9)
Hemiplegia or paralysis	446 (11.7)	14 (1.8) ^a^	14 (6.8) ^b^	0
Cardiovascular disease	979 (25.8)	175 (22.1)	59 (28.6)	8 (36.4)
Diabetes	972 (25.6)	139 (17.6) ^a^	66 (32.0) ^b^	5 (22.7)
Joint & Bone disease	957 (25.2)	162 (20.5) ^a^	92 (44.7) ^a, b^	4 (18.2)
Respiratory disease	538 (14.2)	70 (8.9) ^a^	25 (12.1)	1 (4.5)
Renal disease	422 (11.1)	29 (3.7) ^a^	20 (9.7) ^b^	1 (4.5)
Cancer	183 (4.8)	23 (2.9)	12 (5.8)	0

*Abbreviation*: *LTCI* Long-term care insurance, *BI* Barthel index, *LSGMS* *Lower* Lovett scale of grading muscle strength for at least one lower limb, *LSGMS Upper* Lovett scale of grading muscle strength for at least one upper limb, *LTC* Long-term care

^**a**^ with statistically significant difference compared to beneficiaries without dementia

^**b**^ with statistically significant difference compared to beneficiaries with dementia. *P* value have been Bonferroni corrected. We exclude unsuccessful claimants with dementia for multiple comparison, due to small sample size

The prevalence of chronic diseases was higher in beneficiaries without dementia than that in beneficiaries with dementia, while there were no significant differences in the prevalence of chronic diseases between beneficiaries without dementia and unsuccessful claimants without dementia, except for joint and bone diseases (Table 1). The prevalence of stroke was high in beneficiaries with (38.1%) or without dementia (56.6%), as well as in unsuccessful claimants with (40.9%) or without dementia (52.4%).

### Long-term care needs in the claimants

Beneficiaries with dementia were more likely to be institutionalized than beneficiaries without dementia (Table 1). As shown in Table 2, beneficiaries with dementia needed less nursing care (e.g. care for pressure sores and nasogastric catheterization) and BADL support than beneficiaries without dementia. Nevertheless, there was no group difference in needing support for grooming and continence between beneficiaries with and without dementia. Unsuccessful claimants needed less nursing care and BADL support than beneficiaries with or without dementia (Table 2). Further analysis (Table 3) showed that 14 (63.6%) unsuccessful claimants with dementia and 172 (83.5%) unsuccessful claimants without dementia were disabled in at least one of six basic self-care activities (referring to bathing, dressing, toilet use, transfer, continence, and feeding). Five (22.7%) unsuccessful claimants with dementia and 48 (23.3%) unsuccessful claimants without dementia were disabled in at least two of six basic self-care activities (Table 3).

**Table 2:** Long-term care needs in claimants of the LTCI pilot (*n* = 4810)

Variables	Beneficiaries		Unsuccessful claimants	
	without dementia (***n*** = 3791)	with dementia (***n*** = 791)	without dementia (***n*** = 206)	with dementia (***n*** = 22)
**Needs for nursing treatment, n (%)**				
Pressure sore care	628 (16.6)	37 (4.7) ^a^	5 (2.4) ^a^	0
Dressing change	166 (4.4)	11 (1.4) ^a^	2 (1.0)	0
Nasogastric catheterization	145 (3.8)	11 (1.4) ^a^	1 (0.5) ^a^	0
Urine catheterization	99 (2.6)	6 (0.8) ^a^	2 (1.0)	0
Oxygen therapy	73 (1.9)	4 (0.5) ^a^	5 (2.4) ^b^	0
Colostomy care	32 (0.8)	1 (0.1)	2 (1.0)	0
**Dependent on BADL support, n (%)**				
Bathing	3779 (99.7)	782 (98.9) ^a^	168 (81.6) ^a,b^	14 (63.6)
Climbing stairs	3761 (99.2)	612 (77.4) ^a^	112 (54.4) ^a,b^	4 (18.2)
Mobility	3380 (89.2)	396 (50.1) ^a^	13 (6.3) ^a,b^	0
Toilet use	2825 (74.5)	402 (50.8) ^a^	11 (5.3) ^a,b^	0
Grooming	2384 (62.9)	489 (61.8)	22 (10.7) ^a,b^	0
Dressing	2290 (60.4)	304 (38.4) ^a^	9 (4.4) ^a,b^	0
Transfer	2231 (58.8)	221 (27.9) ^a^	1 (0.5) ^a,b^	0
Bladder	2075 (54.7)	426 (53.9)	14 (6.8) ^a,b^	2 (9.1)
Bowels	1803 (47.6)	396 (50.1)	9 (4.4) ^a,b^	1 (4.5)
Feeding	1197 (31.6)	170 (21.5) ^a^	0 ^a,b^	0

*Abbreviation*: *LTCI* Long-term care insurance, *BADL* Basic activities of daily living

^**a**^ with statistically significant difference compared to successful claimants without dementia

^**b**^ with statistically significant difference compared to successful claimants with dementia

*P* value have been Bonferroni corrected. We exclude unsuccessful claimants with dementia for multiple comparison, due to small sample size

**Table 3:** Number of dependence in six basic self-care activities in LTCI claimants (*n* = 4810)

Number	Beneficiaries						Unsuccessful claimants					
	without dementia (*n* = 3791)			with dementia (*n* = 791)			without dementia (*n* = 206)			with dementia (*n* = 22)		
	N	Percent	Cumulative percent	N	Percent	Cumulative percent	N	Percent	Cumulative percent	N	Percent	Cumulative percent
0	2	0.1	0.1	4	0.5	0.5	34	16.5	16.5	8	36.4	36.4
1	422	11.1	11.2	216	27.3	27.8	124	60.2	76.7	9	40.9	77.3
2	613	16.2	27.4	181	22.9	50.7	34	16.5	93.2	5	22.7	100
3	593	15.6	43	114	14.4	65.1	10	4.9	98.1	0	0	–
4	627	16.5	59.5	93	11.8	76.9	4	1.9	100	0	0	–
5	595	15.7	75.2	60	7.6	84.5	0	0	–	0	0	–
6	939	24.8	100	123	15.5	100	0	0	–	0	0	–

*Abbreviation*: *LTCI* Long-term care insurance; Six basic self-care activities refer bathing, dressing, toilet use, transfer, continence and feeding

### Performance of the Barthel index based on the Rasch model

**Fig. 2:**
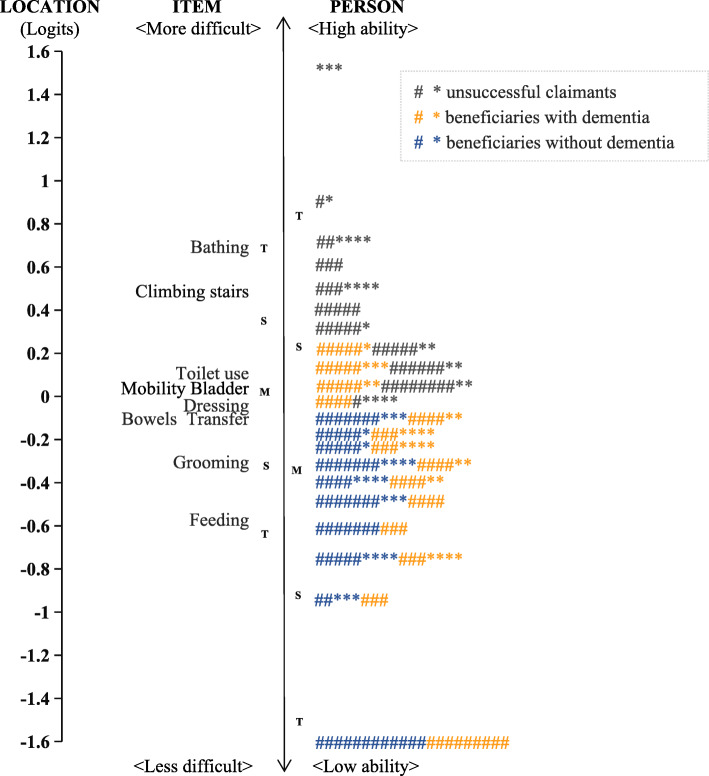
The Wright person-item map of the Barthel Index for assessing beneficiaries with (*n* = 300) or without dementia (*n* = 300) and unsuccessful claimants (*n* = 228). Notes: # = 5 persons, * = 1 person; M = mean person ability or item difficulty, S = one standard deviation, T = two standard deviations. The vertical line is a continuum representing the measures of person ability (right) and item difficulty (left), plotted in logit units. Person ability and item difficulty increase from bottom to top

**Fig. 3:**
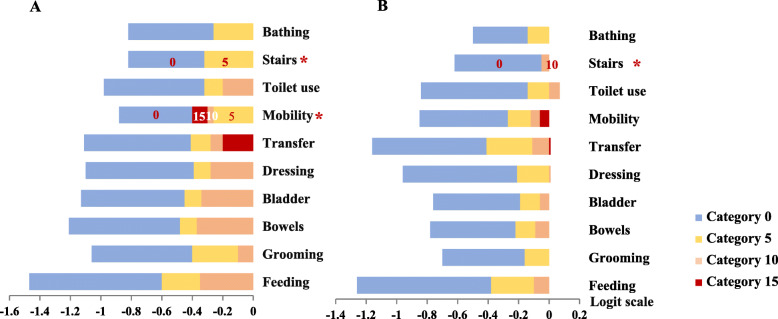
Logit distance between category thresholds for the 10 items of the Barthel Index in beneficiaries without (**a**) or with dementia (**b**). Note: The asterisks indicate disordered or disappeared category thresholds

Accepted unidimensionality, global fit (Table 4) and local fit (Table 5) indicated that the data could be analyzed using a Rasch model. Although item reliability was acceptable for both successful and unsuccessful claimants (0.99–1.0), the person reliability was low (0.39–0.59), indicating narrow ability range of the samples. Figure 2 shows part of unsuccessful claimants and beneficiaries with dementia were co-exist at from − 0.1 to 0.3 logits (around 0.5 to 1 standard deviation of mean person ability), and part of beneficiaries with or without dementia were co-existed at from − 1.6 to − 0.1 logits (around − 2 to 0.5 standard deviation of mean person ability). Figure 3 shows threshold disordering in “climbing stairs” for beneficiaries with dementia and “mobility” for beneficiaries without dementia. A narrow interval was observed among all the adjacent categories of the items (< 1.4 logits).

**Table 4:** Unidimensionality and Global fit statistics for the Barthel index in assessing activities of daily living of the LTCI claimants

Variables	Successful claimants		Unsuccessful claimants (***n*** = 228)	Selected sample (***n*** = 828)
	without dementia (***n*** = 3791)	with dementia (***n*** = 791)		
Cumulative variance explained by Rasch dimension	50.6%	51.5%	43.5%	59.2%
Cumulative variance explained by first contrast	10.8%	13.6%	18.3%	11.4%
Eigenvalue of Rasch dimension	10.3	10.6	7.5	14.5
Eigenvalue of first contrast	2.2	2.8	3.2	2.8
Person reliability	0.39	0.58	0.59	0.69
Item reliability	1.00	0.99	0.99	1.00
Global fit statistics (Log-likelihood χ^2^, *P*)	28,530.53, 0.94	8055.87, 0.65	2822.05, 0.43	8535.46, 0.53
Standardized residuals, mean (SD)	−0.02 (0.84)	−0.01 (0.94)	−0.01 (1.01)	−0.01 (0.91)
Person measure, mean (SD)	−0.82 (0.54)	−0.49 (0.52)	0.28 (0.24)	−0.37 (0.59)

*Abbreviation*: *LTCI* Long term care insurance, *SD* Standard deviation

**Table 5:** Location and item fit statistics for the Barthel Index in assessing activities of daily living of the LTCI claimants

Item	Successful claimants without dementia (*n* = 3791)			Successful claimants with dementia (*n* = 791)			Unsuccessful claimants (*n* = 228)		
	location	Infit		location	Infit		location	Infit	
		MNSQ	ZSTD		MNSQ	ZSTD		MNSQ	ZSTD
Bathing	0.69	1.00	0.1	0.71	1.02	0.2	0.97	0.92	−0.7
Climbing stairs	0.50	1.00	0.1	0.19	0.93	−1.0	0.61	1.07	0.9
Mobility	0.15	0.96	−0.8	−0.14	1.03	0.6	−0.53	1.66	6.1
Toilet use	0.36	0.84	−8.9	0.16	0.79	−4.6	0.05	0.64	−4.0
Grooming	−0.52	1.01	0.4	−0.23	1.10	2.3	0.29	0.52	−6.2
Dressing	0.06	0.90	−4.8	−0.06	0.92	− 1.4	− 0.05	0.78	−2.4
Transfer	0.10	0.93	−2.7	−0.25	0.76	−5.1	−0.69	1.24	2.4
Bladder	−0.27	1.17	6.0	0.13	1.17	3.2	−0.03	1.31	2.9
Bowels	−0.38	1.18	6.7	0.01	1.26	4.4	−0.13	1.28	2.8
Feeding	−0.70	1.02	1.0	−0.52	1.08	1.6	−0.48	0.58	−5.5

*Abbreviation*: *LTCI* Long-term care insurance, *MNSQ* Mean square, *ZSTD* Standardized Z score

## Discussion

To the best of our knowledge, this is the first study to use in-depth analysis of claims data from a pilot city to assess the LTCI pilot in China and provide suggestions of improving the LTCI system. The present study found that stroke and dementia were two common reasons for needing long-term care in LTCI claimants. The Barthel Index is not suitable for assessing and dividing LTCI claimants because of inappropriate items and narrow category responses. A comprehensive assessment and grading system are required, together with needs-led care services provision. The LTCI pilot has played an important role in serving older adults with severe physical disabilities and stimulating needs-led services, although the number of beneficiaries remains relatively small. The eligibility should be expanded gradually based on balance finance solutions.

### High prevalence of chronic diseases in LTCI claimants

Japan reported the top five leading causes of certified need of care, stroke (21.5%), dementia (15.3%), asthenia (13.7%), joint disease (10.9%) and fall-related fracture (10.2%), comprising 71.6% of all causes [14, 15]. Cerebrovascular disease, dementia, and knee disease are also suggested as common diseases requiring long-term care in Korea [16]. In the present pilot, 95.1% of the beneficiaries were over 60 years old, and the prevalence of chronic diseases was high in both beneficiaries and unsuccessful claimants. The high prevalence of stroke (56.6% of beneficiaries without dementia and 52.4% of unsuccessful claimants without dementia) suggests that stroke might be a major reason for needing long-term care in the claimants. The proportion of beneficiaries with dementia (17.3%) in Guangzhou is higher than that in Japan (15.3%). It is possible because of the high prevalence of stroke (38.1%) in beneficiaries with dementia in our samples. The findings also suggest that preventing stroke might decrease future need for long-term care services in Chinese older adults.

### Care needs in the LTCI beneficiaries

The LTCI pilot in China aims to provide BADL support and nursing care related to BADL for disabled older persons [2]. We found that beneficiaries without dementia needed more BADL support services and nursing care than beneficiaries with dementia, while beneficiaries with dementia were more likely to use institutional services. The higher prevalence of chronic diseases in beneficiaries without dementia might have contributed to more nursing care needs. On the contrary, people with dementia may be harder to care for at home, even with good physical function, and therefore more likely to be institutionalized. The findings support that clinical characteristics of claimants might help to predict their care needs.

It is vital to provide social support to prevent institutionalization and ensure that people with dementia can continue to live in the community [17]. In Japan, several community-based care services are provided for patients with dementia, including day services, day rehabilitation, respite services, and group homes [17]. In the pilot, home care provided mainly focused on support for BADL and nursing care. However, beneficiaries with dementia had less need for both of these services than those without dementia, suggesting that care service provision should be established to meet needs, such as dementia-appropriate supports. Consequently, cognitive training, occupational therapy, and dementia care have been added to the services list in the Guangzhou pilot since July 2019 (see Additional file 1). Although further investigation of the feasibility of these new services is needed, the current pilot has been playing an important role in stimulating development of long-term care services, and bridging the gap between demand and supply.

### Unmet needs in unsuccessful claimants

When we counted the number of disabilities in six basic self-care activities (bathing, dressing, toilet use, transfer, continence and feeding) according to the Markov model, 63.6% unsuccessful claimants with dementia and 83.5% unsuccessful claimants without dementia were disable in at least one of six activities in this pilot. Moreover, in this pilot, 22.7% of unsuccessful claimants with dementia and 23.3% of unsuccessful claimants without dementia were disabled in at least two of six basic self-care activities. According to an official report issued by Guangzhou government, of 840,900 insured individuals in the Guangzhou pilot, 10,266 (1.2%) had received LTCI benefits by the end of July 2019 [18]. This number was relatively small and lower than in Japan and Germany, where LTCI provides benefits to 13.5% and 10.5% of the population aged over 65 years, respectively [19]. Our findings suggest there are unmet needs for assistance with BADL in unsuccessful claimants in the pilot. However, China has the world’s largest population of older adults with public sources contributing the largest share to finance old-age [20]. The LTCI eligibility should be expanded gradually based on balance finance solutions.

Interestingly, the unsuccessful claimants were more likely to use institutional services in the pilot. A possible reason is that persons residing in long-term care facilities might have easy access to the application process because most of the long-term care facilities were designated agencies to begin applying for LTCI benefits in the pilot. Policymakers should pay attention to exploring various methods of prompting access to long-term care resources.

### Improving assessment for LTCI claimants

In China, 10 of the 15 long-term care insurance pilots use the Barthel Index to measure functional dependence [2]. Our Rasch analysis found a narrow interval and disordering threshold among the categories in the scale, suggesting that some of the items should be revised, and their categories could be collapsed if the scale is used to assess the functional dependence of long-term care recipients. We also found that it is difficult to divide beneficiaries using the Barthel Index scores, because of their narrow physical ability range. Moreover, there were much overlap in severity of disabilities between beneficiaries with and without dementia, and between unsuccessful claimants and beneficiaries with dementia, when assessed by the scale. Our findings suggest that the Barthel Index is not suitable and sensitive to distinguish LTCI claimants and their care needs.

Persons with dementia might find it harder to access the LTCI, because eligibility for benefits is mainly based on physical impairment (measured by the Barthel Index in most of the pilots), and not cognition or behavioral disturbance. Lack of items to assess cognition and behavioral problems in people with dementia may underestimate their needs [21]. Healthcare professionals and researchers have engaged in active debates about expanding eligibility for persons with dementia because these persons often need time-consuming general supervision [22, 23]. In well-developed LTCI systems, assessments commonly include physical and cognitive function, behavioral symptoms, nursing and special care needs, rehabilitation needs (for musculoskeletal conditions), and social environment, weighted by an algorithm [16, 22, 24, 25]. Policymakers should consider including cognitive and behavioral criteria to ensure people with dementia can obtain the necessary care [26, 27]. This is critical, given the increasing number of people with dementia in China [8].

An efficient and integrated LTCI system should subdivide recipients by individual need and provide appropriate services based on functional status and care needs. For example, care needs and benefits are classified into five levels in Germany [22] and six in Japan [10, 15]. Germany even added a category of general supervision and care (care level 0) in 2013 because people with dementia may maintain a high functional ability but require supervision and care because of behavioral disturbance [22]. A comprehensive assessment and grading system should be developed to determine care level for the current LTCI scheme.

### Limitations

This study had several limitations. First, the limited information about the use of individual services limited in-depth analysis of claimants’ care needs. We therefore estimated claimants’ needs from their medical conditions and physical function. Second, data were only available up to March 2019, so changes following revision to long-term care services in August 2019 were not reflected. Third, not all chronic diseases were correctly coded using the International Classification of Diseases 10th version, so there may have been partial diagnostic coding inaccuracies. A qualitative study would help to assess whether there are inequalities in access to insurance for community-based patients with dementia. Future research is therefore needed to determine how to improve long-term care insurance in developing countries.

## Conclusions

In general, the LTCI pilot plays an important role in serving older adults with severe physical disabilities and stimulating the development of needs-led services, although the beneficiaries remain relatively small. The Barthel Index is not suitable for assessing and dividing LTCI claimants because of inappropriate items and narrow category responses. A comprehensive assessment and grading system are required to ensure particular vulnerable or at-risk populations to obtain necessary care services. The eligibility for LTCI benefits should be expanded gradually based on balance finance solutions. The findings may also be useful for low- or middle-income countries seeking to develop a feasible public LTCI system, especially those with a large elderly population.

## Supplementary Information

**Additional file 1.** Services categorized and provided by the long term care insurance policy in Guangzhou pilot.

## Data Availability

The data that support the findings of this study are available from institutions for applying long-term care insurance but restrictions apply to the availability of these data, which were used under license for the current study, and so are not publicly available.

## Abbreviations

LTCI: Long-term care insurance
BADL: Basic activities of daily living
