# Supplementary material for: Health status, care needs, and assessment for beneficiaries with or without dementia in a public long-term care insurance pilot in Guangzhou, China
Source: BMC Health Serv Res. 2020 Dec 7;20:1127. doi: 10.1186/s12913-020-05965-1 (PMC7720481; doi:10.1186/s12913-020-05965-1)
Supplement: Supplementary file 1 — Additional file 1. Services categorized and provided by the long term care insurance policy in Guangzhou pilot. [file 12913_2020_5965_MOESM1_ESM.doc]

**Additional file 1** Services categorized and provided by the long term care insurance policy in Guangzhou pilot

| **ADL support** | **Medical & Nursing treatment** |
| --- | --- |
| **IADL assistance & Environmental safety**  Cleaning room/bathroom and ensure the safety  Cleaning towel/wash basin/toilet  Make the bed  Ensuring the safety of room, adding handrails and bed rails as needed  Ensuring the safety of outdoor activities | **Nursing treatment**  Family visit  Oxygen Therapy (low flow)  Oxygen Therapy (middle flow)  Oxygen Therapy (high flow)  Aspiration of sputum  Diabetic feet care  Nasogastric Tube Insertion  Dressing change (>50cm²)  Dressing change (31-50cm²)  Dressing change (16-30 cm²)  Dressing change (<15 cm²)  Physical hypothermy  Urinary Catheterization care  Decreasing flatulence by rectal tube  Enema  Colostomy care  Bladder irrigation  Swallowing Function Training **^a^**  Oral care **^a^**  Perineal irrigation **^a^** |
| **Bathing**  Tepid water sponge bath/bathing assistance  Shampooing hair in bed |
| **Personal hygiene (Grooming)**  Cleaning face, combing, and oral hygiene  Cleaning hand and foot  Shaving and hair cut  Cleaning perineal and perianal |
| **Dressing**  Change clothes |
| **Feeding**  Tube feeding  Feeding |
| **Excretion (Bladder, Bowels and Toilet use)**  Using bedpan  Incontinence care  Help to relieve urine retention  Help to relieve intestine flatulence or constipation  Fecal impaction care | **Rehabilitation ^a^**  Joint training  Kinesiotherapy  Balance training  Hand function exercise  Partial body weight support system training  Electric standing bed training  Comprehensive training for hemiplegic limb  Comprehensive training for paraplegic limb |
| **Locomotion assistance (Mobility, Transfer and Climbing stairs)** | **Cognitive function training ^a^** |
| **Bedridden care**  Pressure ulcer prevention  Help to change position and shoot back  Help to prevent lung infections  Assisting in limb functional activities | **Occupational therapy ^a^** |
| **Assisting in safe medication administration** |  |
| **Sleeping hygiene** |  |
| **Pay attention to psychological needs**  **Dementia care ^a^**  Disinhibition care  Wandering behavior care/elope prevention  Aggressive behavior care  Keeping language function  Keeping orientation function  Keeping motion function |  |

Abbreviation: ADL, activities of daily living; IADL, instrumental activities of daily living.

^a^ new items added since August 2019.
